# Impact of spread through air spaces (STAS) and lymphovascular invasion (LVI) on prognosis in NSCLC: a comprehensive pathological evaluation

**DOI:** 10.1007/s13304-025-02170-9

**Published:** 2025-04-09

**Authors:** Can Kutlay, Selim Şakir Erkmen Gülhan, Leyla Nesrin Acar, Muhyettin Aslan, Fatma Benli Tanrıkulu

**Affiliations:** 1 Ankara Etlik City Hospital, Ankara, Turkey; 2 Ankara Atatürk Sanatoryum Education and Research Hospital, Ankara, Turkey; 3 İstanbul Çam Sakura City Hospital, Istanbul, Turkey

**Keywords:** Thoracic surgery, Lung cancer, Spread through air spaces, Lymphovascular invasion

## Introduction

Lung carcinoma is the most common cause of death among cancers [1]. Although age, gender, general performance, tobacco use, and comorbidities play a role in prognosis, the most significant factor is the TNM stage [2]. In the current 8th staging, tumor size and pleural invasion are evaluated in the T category, yet spread through air spaces (STAS) of the tumor, and lymphovascular invasion (LVI) are independent pathological features that are not.

STAS is an invasion pattern defined by malignant cells or cell populations in the alveolar spaces next to the tumor border [3]. STAS has three morphological types: micropapillary clusters, solid, and single cells. The World Health Organization (WHO) lung cancer classification identifies STAS as contributing to a poor prognosis by elevating the risk of recurrence, particularly in limited resections performed for early-stage adenocarcinoma [4].

LVI is identified by the presence of a tumor in the endothelial-lined lymphatic or blood vessels near the neoplasm. Cases with LVI have a higher risk of nodal spread and distant organ metastasis, leading to a poorer outcome due to vascular and lymphatic invasion [5, 6].

Cases with LVI showed a higher likelihood of nodal spread and distant organ metastasis. The National Comprehensive Cancer Network (NCCN) classifies vascular invasion as a high-risk condition in its non-small cell lung cancer guideline, and it suggests considering adjuvant chemotherapy for patients with vascular invasion [7].

This study aimed to reevaluate the pathology specimens of individuals who underwent surgery for non-small cell lung cancer, examining STAS and LVI. Our focus was on understanding the impact of these factors on the rates of recurrence and overall survival.

## Patients and methods

The T.C. Sağlık Bilimleri Üniversitesi Ankara Keçiören Eğitim ve Araştırma Hastanesi Institutional Review Board approved this study (2012-KAEK-15/2454). The study retrospectively collected data from patients who underwent surgery at our clinic with diagnoses of squamous cell carcinoma, adenocarcinoma, and adenosquamous carcinoma between January 2012 and January 2016. Patients with a history of prior cancer, distant organ metastasis at the time of diagnosis, mortality due to early postoperative complications, loss to follow-up post-surgery, or unavailability of medical records were excluded from the study.

Postoperative pathological staging was conducted using the 8th edition of the TNM staging system. Local recurrence was defined as the detection of the same tumor type within 2 years post-operation in adjacent lung tissue, the ipsilateral bronchus, main bronchus, or ipsilateral interlobar, hilar, or mediastinal lymph nodes, identified histologically or radiologically.

The pathology department reevaluated the specimens for histopathological diagnosis, STAS, and LVI. They examined lung tissue adjacent to the tumor under × 100, × 200, and × 400 magnifications, following the criteria of Kadota et al., Warth et al., and Ohe et al. [3, 8, 9]. To strengthen the validity, all specimens were reviewed by another pathologist.

**Fig. 1:**
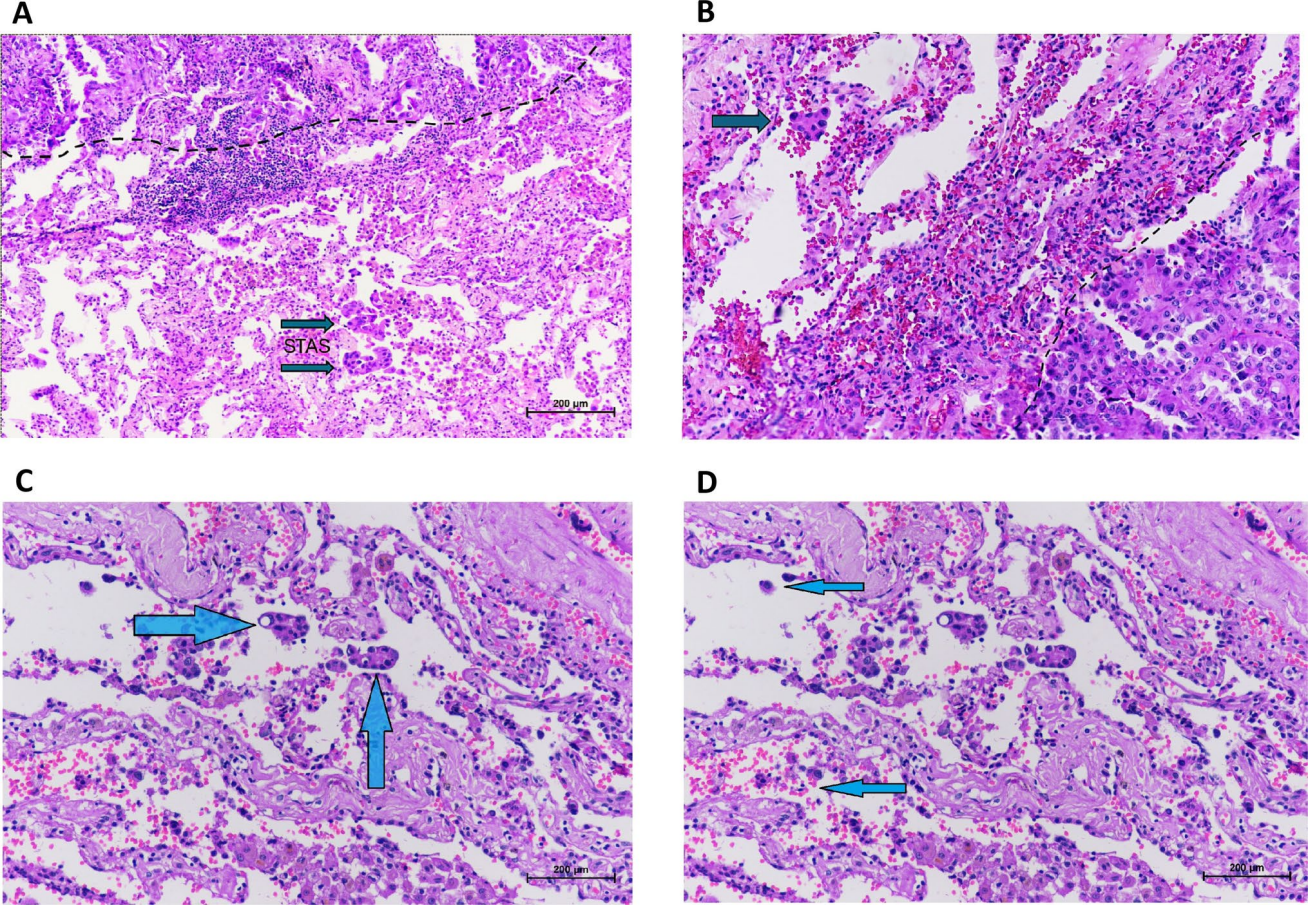
Morphological Features of STAS Invasion in Adenocarcinoma (Hematoxylin & Eosin, × 100 in **A**, × 200 in **B**, **C**, **D**). A, Multiple sites of STAS are observed, with two of them indicated by arrows. B, a higher magnification view shows a small cluster within the alveolar spaces, with the tumor border shown with a dashed line; STAS can be seen in the neighboring alveolar space. C, the arrow-marked areas indicate single-cell STAS, consisting of scattered, discohesive single tumor cells freely floating within air spaces. D, the arrow-marked regions highlight micropapillary-type STAS, consisting of papillary structures without central fibrovascular cores

STAS is defined as micropapillary structures consisting of papillary structures without central fibrovascular cores, solid nests or tumor island filing air spaces, and single cells consisting of scattered discohesive single cells. Tumor cells were identified in at least one airspace beyond the tumor edge, with at least two separate airspace clusters (Fig. 1). Artifacts were excluded based on the original definition. Artifacts included mechanically induced tumor floaters, which appeared randomly, often at the edge of the tissue section or out of the plane of the section. Jagged edges of tumor cell clusters resulted from tumor fragmentation or cuts during specimen processing. Isolated tumor clusters located far from the tumor, rather than spreading continuously from the tumor edge, and linear strips of cells lifted off alveolar walls were also identified as artifacts.

Immunohistochemistry using CK7, CD68, and p53 stains was applied in selected sections to distinguish STAS from alveolar macrophages. Lymphovascular invasion (LVI) was defined as tumor cells within endothelial-lined spaces, including lymphatic and vascular channels.

We conducted statistical analyses using RStudio (version 2021.0.0, PBC), with the tidyverse package for data organization. Univariable Cox regression analysis was performed using the survival package to identify potential factors associated with RFS and OS. Significant factors from the univariable analysis were then included in a multivariable Cox regression model to evaluate their independent effects. Kaplan–Meier survival curves were generated to visualize survival distributions. The Chi-square test was used to evaluate the association of STAS and LVI with clinicopathological parameters. Data visualization was performed with the ggplot2 package. A *p* value of < 0.05 was considered statistically significant for all analyses.

## Results

We identified 352 patients who met the study criteria, of whom 86.3% were male, with a mean age of 59.9 years (range 16–85). All patients underwent pulmonary resection and lymph node dissection. The surgical procedures and their corresponding percentages are presented in Table 1.

**Table 1:** Distribution of resection types in the study population

Resection type	*n*	%
Upper lobectomy	190	54
Lower lobectomy	109	31
Bilobectomy inferior	23	6.6
Pneumonectomy	10	2.8
Middle lobectomy	10	2.8
Bilobectomy superior	7	2
Segmentectomy	3	0.8
Total	352	100

*n* number of patients, % Percentage of total cases

The diagnoses of the patients were 175 (49.7%) squamous cell carcinoma, 165 (46.9%) adenocarcinoma, and 12 (3.4%) adenosquamous carcinoma. The average tumor size across cases was 3.67 cm. We categorized the cases into four groups based on lymph node metastasis. Lymph node status distribution among cases revealed the following percentages: N0 (71.5%), N1 (18.7%), N2 (6.5%), and multiple N2 (3.1%). Pleural invasion was observed in 47.4% of cases.

Table 2 presents the results of a univariable Cox regression analysis evaluating clinical parameters associated with RFS and OS. Age, lymph node invasion, stage, and STAS demonstrated statistically significant effects on survival, while tumor size, pleural invasion, and lymphovascular invasion did not. This may be due to the uneven distribution of disease stages, as 44.6% of patients were stage I, along with the influence of other strong prognostic factors, which may have overshadowed the effect of tumor size on survival.

**Table 2:** Univariate analysis of clinical parameters and their association with RFS and OS

	RFS		OS	
	HR (%95 CI)	*p* value	HR (%95 CI)	*p* value
Gender				
Male (ref)	1		1	
Female	1.05 (0.61–1.81)	0.857	0.869 (0.505–1.494)	0.61
Age	**1.03 (1.009–1.054)**	**0.004**	**1.03 (1.016–1.063)**	** < 0.001**
Operation side				
Right (ref)	1		1	
Left	1.25 (0.86–1.80)	0.23	1.174 (0.818–1.685)	0.383
Diagnosis				
Adenocarcinoma (ref)	1		1	
Squamous cell carcinoma	0.93 (0.64–1.36)	0.74	1.034 (0.716–1.493)	0.857
Adenosquamous carcinoma	1.00 (0.36–2.77)	0.995	1.039 (0.376–2.867)	0.941
Tumor size	1.04 (0.94–1.15)	0.427	1.034 (0.935–1.144)	0.504
Lymph node				
Negative (ref)	1		1	
N1 positive	1.32 (0.81–2.12)	0.25	1.309 (0.819–2.090)	0.259
N2 positive	**2.64 (1.46–4.77)**	** < 0.001**	**2.315 (1.283–4.177)**	**0.005**
Multiple N2 positive	**5.15 (2.46–10.77)**	** < 0.001**	**3.790 (1.824–7.875)**	** < 0.001**
Pleura				
Negative invasion (ref)	1		1	
Positive invasion	1.17 (0.81–1.69)	0.4	1,112 (0.775–1.595)	0.564
Stage				
IA (ref)	1		1	
IB	0.82 (0.43–1.55)	0.55	0.661 (0.357–1.222)	0.186
IIA	0.84 (0.33–2.15)	0.72	0.697 (0.278–1.745)	0.440
IIB	1.44 (0.81–2.57)	0.20	1.119 (0.643–1.946)	0.688
IIIA	**2.40 (1.30–4.40)**	**0.004**	**1.870 (1.043–3.349)**	**0.035**
IIIB	**5.18 (2.03–13.18)**	** < 0.001**	**3.365 (1.341–8.442)**	**0.009**
Postoperative treatment				
Negative (ref)	1		1	
Chemotherapy	1.09 (0.71–1.66)	0.67	1.017 (0.674–1.533)	0.936
Chemo-Radiotherapy	1.62 (0.90–2.90)	0.10	1.419 (0.797–2.527)	0.234
Radiotherapy	2.97 (0.91–9.7)	0.07	2.572 (0.792–8.358)	0.116
STAS				
Negative (ref)	**1**		1	
Positive	**5.83 (3.43–9.92)**	** < 0.001**	**4.649 (2.810–7.690)**	** < 0.001**
Lymphovascular invasion				
Negative (ref)	**1**		1	
Positive	**1.49 (1.018–2.180)**	**0.039**	1.391 (0.956–2.022)	0.083

*HR* Hazard ratio, *CI* confidence interval, *RFS* recurrence-free survival, *OS* overall survival. Statistically significant *p* values (*p* < 0.05) are in bold

STAS was present in 208 of 352 cases (59.1%), with a higher prevalence in adenocarcinoma (65%) compared to squamous cell carcinoma (54.3%). It was significantly more frequent in adenocarcinoma (*p* = 0.04). STAS was associated with pleural invasion, stage, local recurrence, and distant metastases but showed no significant relationship with tumor size, lymph node metastasis, or lymphovascular invasion. Table 3 summarizes these relationships with various pathological features.

**Table 3:** Association between STAS and pathological parameters

	STAS (−)	STAS (+)	*p* value
Average tumor size	3.59 cm	3.7 cm	0.60
Lymph node metastasis			
n0	108 (%77.1)	141 (%67.8)	0.14
n1	24 (%17.1)	48 (%19.7)	
n2	6 (%4.3)	17 (%8.7)	
Multiple n2	2 (%1.4)	9 (%4.3)	
Stage			
I	**71 (%50.7)**	**85 (%40.8)**	**0.03**
II	**53 (%37.8)**	**76 (%36.5)**	
III	**16 (%11.4)**	**47 (%22.5)**	
Pleural invasion			
Positive	**56 (%40)**	**108 (%51.9)**	**0.03**
Negative	**84 (%60)**	**100 (%48.1)**	
Lymphovascular invasion			
Positive	46 (%32.9)	72 (%34.6)	0.70
Negative	94 (%67.1)	131 (%65.4)	
Relapse			
Negative	**126 (%90)**	**92 (%44.2)**	** < 0.001**
Local relapse	**10 (%7.1)**	**68 (%32.7)**	
Distant metastasis	**4 (%2.9)**	**48 (%23.1)**	

*STAS* Spread through air spaces, Statistically significant *p* values (*p* < 0.05) are in bold

LVI was present in 119 cases (34.6%), with a similar distribution across histological types: 30.8% in adenocarcinoma, 37.7% in squamous cell carcinoma, and 40% in adenosquamous carcinoma. Lymph node involvement was observed in 7.4% of patients without LVI, compared to 69.7% of those with LVI at N1 or N2 stations. The presence of LVI significantly increased the likelihood of lymph node metastasis and higher staging (*p* < 0.001 for both). Although local recurrence was more frequent in LVI-positive patients than in LVI-negative patients, this difference was not statistically significant (*p* = 0.08) (Table 4).

**Table 4:** Association of lymphovascular invasion (LVI) with clinicopathological factors

	LVI (−) (*n*, %)	LVI (+) (*n*, %)	*p* value
Mean tumor size	3.63 cm	3.7 cm	0.72
Lymph node metastasis			
N0	213 (92.6%)	36 (30.3%)	** < 0.001**
N1	13 (5.7%)	53 (44.5%)	
N2	4 (1.7%)	19 (16%)	
Multiple N2	0 (0%)	11 (9.2%)	
Pathological stage			
Stage I	135 (58.7%)	21 (17.6%)	** < 0.001**
Stage II	75 (32.6%)	55 (46.2%)	
Stage III	20 (8.7%)	43 (36.1%)	
Pleural invasion			
Present	110 (47.8%)	64 (53.8%)	0.90
Absent	120 (52.2%)	55 (46.2%)	
Recurrence			
None	153 (66.5%)	65 (54.6%)	0.07
Local	45 (19.5%)	33 (27.7%)	
Distant metastasis	32 (14%)	21 (17.6%)	

*LVI* lymphovascular invasion, statistically significant *p* values (*p* < 0.05) are in bold

Confounding factors such as STAS, LVI, and age were included in the multivariable analysis, along with pleural invasion and tumor size, as they are known to affect prognosis. LVI and lymph node metastasis have a strong association, with LVI potentially contributing to lymph node metastasis, so lymph node metastasis and stage were excluded. The multivariable analysis identified LVI, STAS, and age as significant factors influencing both RFS and OS, as shown in Table 5.

**Table 5:** Multivariate analysis of clinical parameters and their association with RFS and OS

	RFS		OS	
	HR (%95 confidence interval)	*p* value	HR (%95 confidence interval)	*p* value
Age	**1.033 (1.010–1.056)**	**0.003**	**1.041 (1.018–1.065)**	** < 0.001**
Tumor size	1.026 (0.914–1.152)	0.659	1.014 (0.905–1.137)	0.799
Pleura				
Negative invasion (ref)	1		1	0.872
Positive invasion	0.979 (0.655–1.462)	0.918	0.968 (0.655–1.431)	
STAS				
Negative (ref)	**1**		1	** < 0.001**
Positive	**5.804 (3.404–9.898)**	** < 0.001**	**4.595 (2.770–7.622)**	
Lymphovascular invasion				
Negative (ref)	**1**		**1**	**0.002**
Positive	**1.718 (1.165–2.531)**	**0.006**	**1.554 (1.062–2.274)**	

*HR* Hazard ratio, *CI* Confidence interval, *RFS* Recurrence-free survival, *OS* Overall survival

Statistically significant *p* values (*p* < 0.05) are in bold

### RFS

Patients were monitored for 5 years post-operation. During this period, 78 (22.1%) experienced local recurrence, and 52 (14.7%) developed distant metastases. Patients with STAS had a significantly lower 5 year RFS rate (49%) than those without STAS (90.7%). Among the studied parameters, STAS was the strongest predictor of poorer 5 year RFS and was associated with a significant reduction in RFS across all histological types (*p* < 0.001). The cases were grouped by stage, and STAS negatively impacted RFS and OS across all stages (Table 6**)**.

**Table 6:** Impact of STAS on RFS and OS by stage

Stage	RFS		OS	
	HR (95% CI)	*p* value	HR (95% CI)	*p* value
Stage I	5.13 (2.25–11.65)	** < 0.001**	4.09 (1.88–8.86)	** < 0.001**
Stage II	5.43 (2.27–12.96)	** < 0.001**	4.29 (1.90–9.68)	** < 0.001**
Stage III	6.51 (1.96–21.57)	** < 0.001**	4.88 (1.49–16.03)	**0.0012**

*HR* hazard ratio, *CI* confidence interval, *RFS* recurrence-free survival, *OS* overall survival

Statistically significant *p* values (*p* < 0.05) are in bold

LVI was associated with a lower 5 year RFS rate, at 56.3%, compared to 67.8% in patients without LVI. It significantly reduced RFS in both univariable and multivariable analyses. Among histological types, adenocarcinoma had significantly worse RFS (*p* = 0.03), while squamous cell carcinoma and adenosquamous carcinoma showed no significant difference.

### OS

**Fig. 2:**
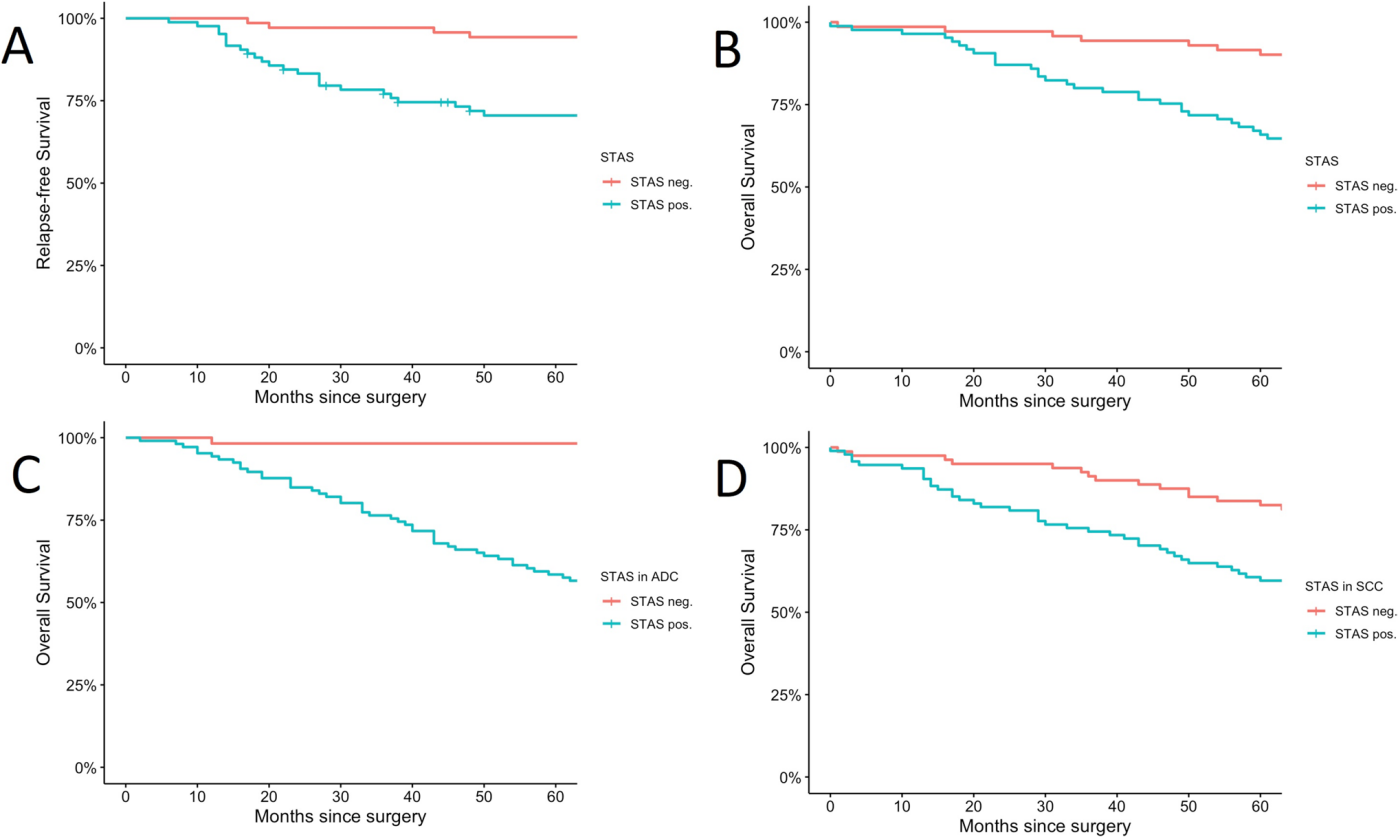
Kaplan–Meier survival curves illustrating the impact of spread through air spaces (STAS) on survival outcomes in patients undergoing surgery. **A** Relapse-free survival (RFS) in Stage I (*p* < 0.001): 1 year: 100% vs. 88.3%, 3 year: 96.8% vs. 71.8%, 5 year: 93% vs. 60%. **B** Overall survival (OS) in Stage I (*p* < 0.001): 1 year: 99.4% vs. 91.4%, 3 year: 96% vs. 76.1%, 5 year: 90.1% vs. 65.9%. **C** OS in adenocarcinoma (ADC) (p = 0.003): 5 year: 98.2% vs. 42.9%. **D** OS in squamous cell carcinoma (SCC) (p < 0.001): 5 year: 82.5% vs. 60%. Survival rates are shown for STAS (−) vs. STAS ( +)

During the 5 year follow-up, 101 patients (28.6%) died. OS was 58.6% in patients with STAS, compared to 89.3% in those without. While LVI, age, lymph node metastasis, and stage were associated with poorer survival, STAS emerged as the most significant factor negatively impacting OS and was associated with a significant reduction in OS across all histological types. In squamous cell carcinoma, the survival was lower in patients with STAS compared to those without, and the same trend was observed in adenocarcinoma and adenosquamous carcinoma. The impact of STAS on OS was statistically significant (*p* < 0.001, *p* = 0.003, *p* < 0.001, respectively) (Fig. 2).

**Fig. 3:**
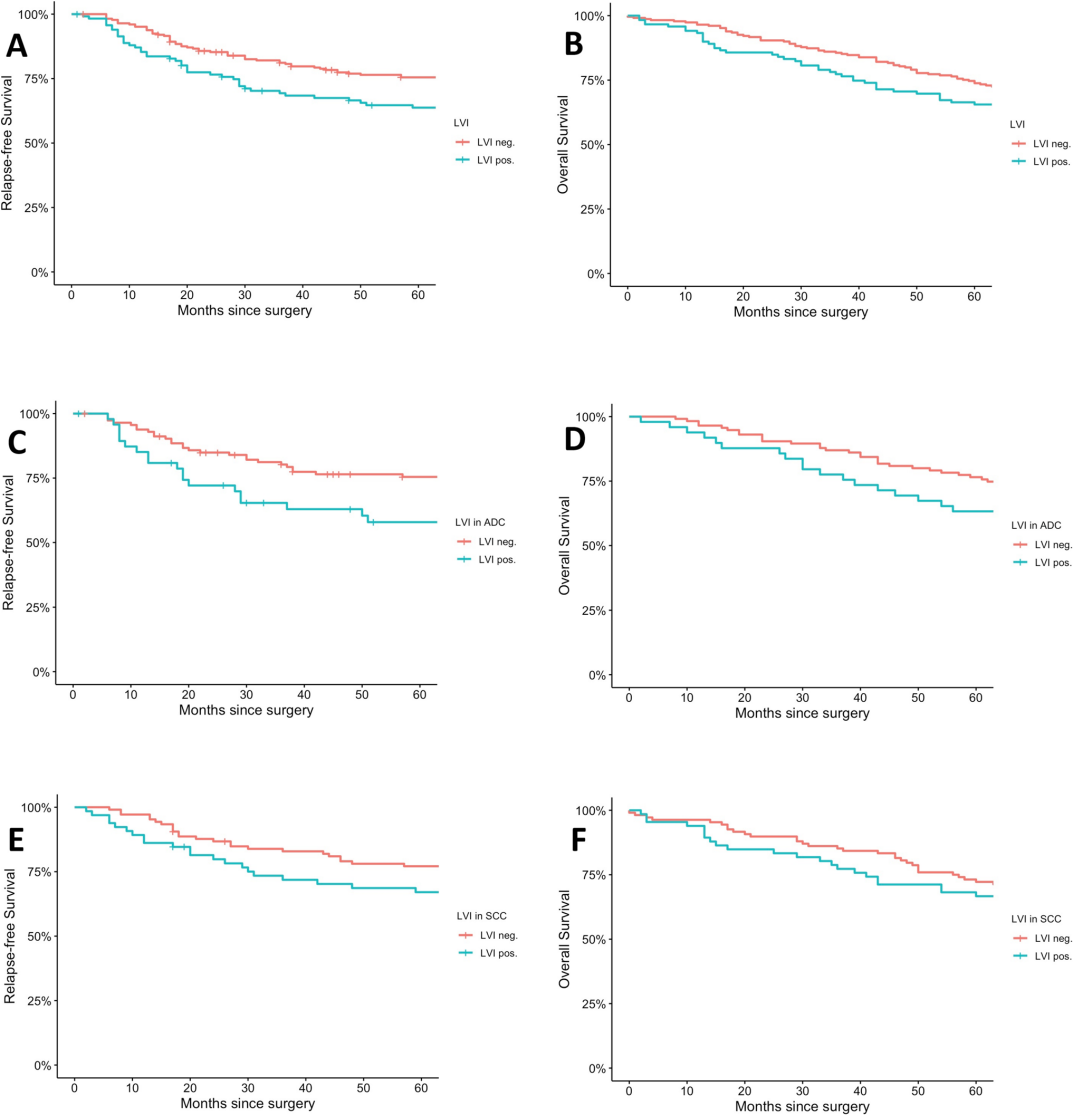
Kaplan–Meier survival curves assessing the prognostic significance of lymphovascular invasion (LVI) in patients with surgical resection. **A** Relapse-free survival (RFS) in all patients (*p* = 0.006): 5 year: 67.8% vs. 56.3%. **B** Overall survival (OS) in all patients (*p* = 0.002): 5 year: 73.5% vs. 65.5%. **C** RFS in adenocarcinoma (ADC) (*p* = 0.03): 5 year: 64.3% vs. 44.9%. **D** OS in ADC (*p* = 0.04): 5 year: 76.5% vs. 63.3%. **E** RFS in squamous cell carcinoma (SCC): 5 year: 73.4%. vs 63.6% and **F** OS in SCC: 5 year: 71.4% vs. 66.7%, were not statistically significant. Survival rates are shown for LVI (−) vs. LVI ( +)

The 5 year OS rate was 65.5% in patients with LVI, compared to 73.5% in those without. Multivariate analysis revealed that LVI significantly reduced OS. When patients were grouped by histological type, LVI was found to decrease OS by 1.76 times in those with adenocarcinoma (HR 95% CI 1.01–3.08, *p* = 0.04). However, no statistically significant relationship was observed between LVI and OS in patients with squamous cell carcinoma or adenosquamous carcinoma (Fig. 3).

### STAS subtypes

Solid and micropapillary STAS were grouped as solid clusters due to their morphological similarities. STAS was observed as solid clusters in 53.3% of cases and as single cells in 46.7%.

**Fig. 4:**
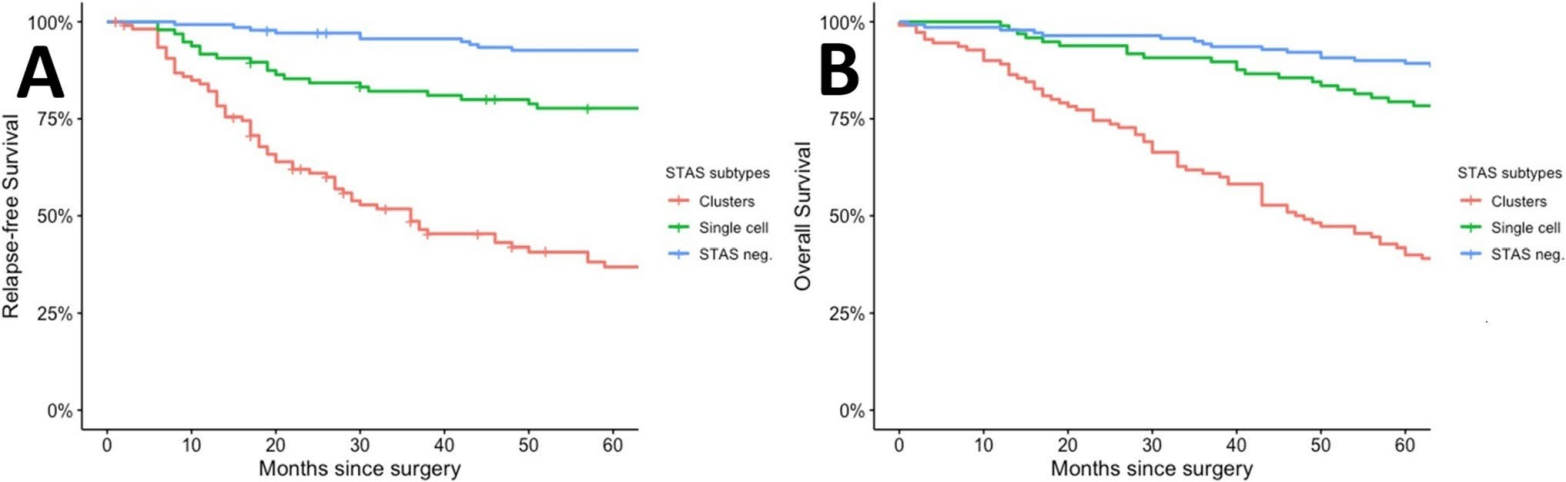
Relapse-Free Survival (RFS) and Overall Survival (OS) by STAS Subtypes. **A** 5 year RFS: 71.1% (single-cell STAS) vs. 26.1% (solid-type STAS). Both significantly reduced RFS (*p* = 0.0018, *p* < 0.001). Solid-type STAS decreased RFS 4.06 × more than single-cell (HR 2.55–6.45, *p* < 0.001). **B** 5 year OS: 79.4% (single-cell STAS) vs. 39.6% (solid-type STAS). Both significantly reduced OS (*p* = 0.012, *p* < 0.001). Solid-type STAS decreased OS 3.74 × more than single-cell (HR 2.36–5.90, *p* < 0.001)

The 5 year RFS was 71.1% for single-cell STAS and 26.1% for solid-type STAS, with both subtypes significantly reducing RFS. Similarly, the 5 year OS was 79.4% for single-cell STAS and 39.6% for solid-type STAS, both significantly reducing OS compared to patients without STAS (Fig. 4).

## Discussion

Many studies have observed malignant cells or cell clusters adjacent to the cancer margin in alveoli and researchers previously used terms like alveolar growth pattern or tumor islands to describe this phenomenon. Kadota and colleagues coined the term “spread through air spaces” (STAS) to define this phenomenon. Their research established a correlation between STAS and elevated recurrence rates, as well as a poorer prognosis, especially in cases of squamous cell carcinoma and adenocarcinoma [3, 10].

Blaauwgeers et al. proposed that what is often identified as STAS may actually be tumor cell fragments resulting from biopsy procedures, surgical manipulation, and pathological tissue handling, including cutting techniques during sample preparation [11]. They argued that the survival of individual tumor cells in air spaces is biologically implausible. In contrast, Yagi et al. conducted three-dimensional histologic reconstruction, immunohistochemistry, and multiplex immunofluorescence analyses to study STAS in lung adenocarcinomas [12]. Their findings demonstrated that tumor cells within STAS actively co-opt preexisting blood vessels, highlighting STAS as a genuine invasive mechanism. This suggests that the tumor cells detach from the main tumor, migrate through air spaces, and reattach to the alveolar walls through vessel co-option, allowing them to survive and grow.

Since 2015, multiple studies have established STAS as an independent pathological prognostic factor [10, 13–16]. In our analysis of patients who underwent surgery for NSCLC over four years, STAS emerged as the most significant predictor of prognosis among pathological parameters, including tumor size, pleural invasion, lymph node metastasis, pathological stage, and LVI. Furthermore, stratification by stage and histological subtype demonstrated a consistently higher recurrence rate in STAS-positive cases across all groups, emphasizing its role in disease progression.

Since 2015, multiple studies have established STAS as an independent pathological prognostic factor [10, 13–16]. In our analysis of patients who underwent surgery for NSCLC over 4 years, STAS emerged as the most significant predictor of prognosis among pathological parameters, including tumor size, pleural invasion, lymph node metastasis, pathological stage, and LVI. Furthermore, stratification by stage and histological subtype demonstrated a consistently higher recurrence rate in STAS-positive cases across all groups, underscoring its critical role in disease progression.

Xie and colleagues reported disparities among STAS subtypes [17]. In their study, STAS-negative patients had better survival, while the micropapillary cluster and solid subtypes were identified as independent prognostic factors in multivariate analysis. However, they did not find similar results for the single-cell subtype. Oppositely, we found that single-cell STAS was associated with significantly lower RFS and OS in both adenocarcinoma and squamous cell carcinoma compared to STAS-negative patients. Additionally, we observed that the solid-type STAS had a significantly worse prognosis than both STAS-negative and single-cell STAS cases. This raises the question of whether some cases of single-cell STAS might represent false positives, potentially contributing to differences in prognostic impact. These false positives may arise due to observer bias by pathologists or artifacts introduced during tissue preparation, such as mechanical displacement of tumor cells during specimen handling, sectioning, or staining. Alternatively, single-cell STAS may exhibit varying degrees of aggressiveness compared to the solid type, which could explain the observed differences in outcomes. Larger multicenter studies incorporating standardized pathological assessment techniques may help establish more consistent and reproducible criteria for identifying STAS subtypes and their prognostic significance.

Patients exhibiting the STAS invasion pattern were associated with higher stages, indicating more aggressive tumor behavior. Numerous studies have correlated STAS with tumor size, lymph node metastasis, and pleural invasion [9, 14–16, 18]. In their investigation, Liu and colleagues focused on Twist and Slug molecules, which reduce the expression of the adhesion molecule E-cadherin. They linked these molecules to disruptions in tumor cells and metastases, establishing a correlation with STAS. They proposed that the molecular mechanism of STAS might involve the overexpression of Twist and Slug [14]. Further studies are needed to explore the underlying molecular mechanisms of STAS and its association with other pathological factors, such as LVI. Investigating the interplay between these factors and their impact on prognosis could provide valuable insights into tumor progression and potential therapeutic targets.

Identifying STAS in frozen sections during surgery can help guide decisions about the extent of resection, as positive STAS in limited resections is associated with a higher risk of local recurrence [3, 19]. The sensitivity of detecting STAS in frozen sections ranges from 47.9 to 71% [19, 20]. Although both studies reported high specificity, the variable, and generally low sensitivity—largely due to subjective interpretation—limits the routine use of frozen sections in current practice. However, informing the surgeon about detected STAS can still provide valuable guidance during surgery.

LVI independently impacted the prognosis in adenocarcinoma patients, increasing lymph node metastasis and raising the stage. Five year follow-ups revealed a higher frequency of local recurrence and distant organ metastasis in cases with detected lymphovascular invasion. Few retrospective analyses in non-small cell lung cancers (NSCLCs) have also determined LVI as an independent factor for poor prognosis [21–23].

Our study has a retrospective, single-center design, which may affect the generalizability of the findings. We found STAS as an independent pathological factor that negatively influences prognosis in both adenocarcinoma and squamous cell carcinoma in Stage I, II, and III patient groups. The presence of LVI was associated with reduced RFS and OS. When analyzed by histological type, the adenocarcinoma group showed significantly decreased RFS and OS in patients with LVI. While our results suggest a potential role for STAS and LVI in refining prognostic assessments, further large-scale, multicenter studies are needed to evaluate whether STAS should be incorporated into the TNM staging system.
